# Apolipoproteins L involvement in immunity

**DOI:** 10.70962/jhi.20250259

**Published:** 2026-03-19

**Authors:** Etienne Pays

**Affiliations:** 1 Institute of Molecular Biology and Medicine, University of Brussels, Gosselies, Belgium

## Abstract

Apolipoproteins L (APOLs) are membrane-associated proteins involved in both resistance to pathogens, such as APOL1-mediated killing of African trypanosomes or APOL3-mediated lysis of intracellular bacteria, and induction of diseases, like APOL1-mediated nephropathy or APOL2-mediated liver fibrosis. Accumulating evidence points to APOLs controlling membrane dynamics linked to immunity. APOL1 and APOL3 are induced by inflammatory signalling and play key roles in the initiation and termination of inflammation by promoting the traffic of Golgi-derived membranes involved in STING activation, as well as mitochondrial membrane fission and fusion involved in auto/mitophagy. APOL2, or murine mAPOL8, is required for profibrotic vesicle exocytosis, whereas mAPOL9 triggers bacterial membrane budding linked to gut immunity control. In dendritic cells, APOL3 or the APOL3-like mAPOL7C promote megapore formation in phagosomal membranes, allowing antigen cross-presentation and apoptosis, both probably linked to cardiolipin solubilization. In adipocytes, mAPOL6 controls inflammation-linked lipid droplets dynamics. Through their membrane-remodeling activities, APOLs participate in the control of infection by bacteria, viruses, and parasites. Thus, natural APOLs mutations represent inborn errors of immunity.

## Teaser

Etienne Pays proposes that the lipid-interacting proteins of the APOL families specifically control cellular membrane dynamics involved in processes linked to immunity, like antigen presentation, inflammation, mitophagy, and apoptosis. Dysfunctions in these activities are responsible for diseases such as kidney disease, liver fibrosis, or increased sensitivity to trypanosome infection.

## Introduction

**Figure 1.**
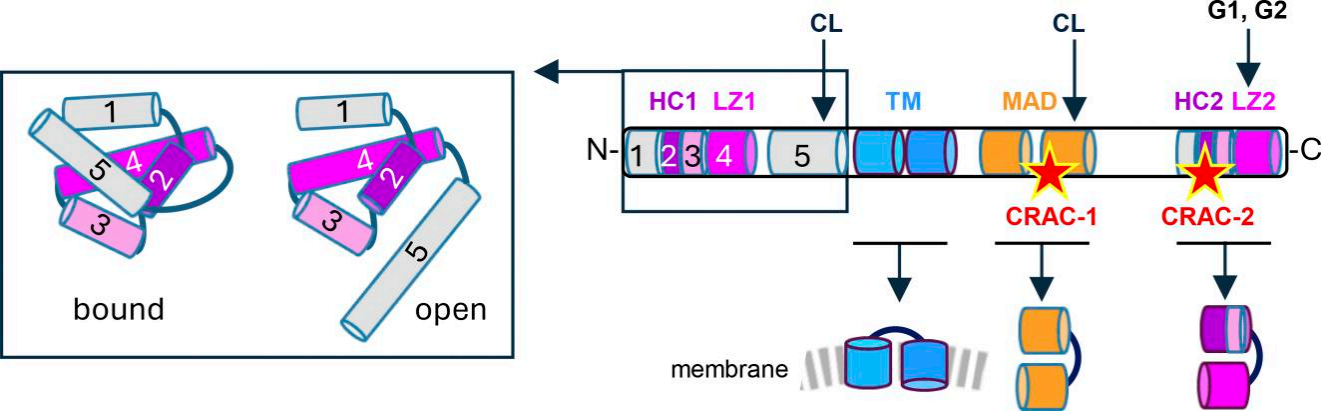
**Structural features of APOL1.** This scheme represents the secreted APOL1 isoform after removal of its specific N-terminal signal peptide. It also summarizes the structure of the other human APOLs or mAPOLs, none of which appears to be secreted. The colored cylinders represent different α-helices, some of which are numbered (4). HC1, HC2 = hydrophobic clusters; LZ1, LZ2 = leucine zippers; CL = potential cardiolipin-binding sites; MAD = membrane-addressing domain; CRAC-1, CRAC-2 = cholesterol recognition amino acid consensuses (represented by red stars); TM = potential transmembrane double-stranded hairpin helix. Whereas transmembrane APOL1 insertion strictly requires acidic conditions, such insertion can occur at neutral pH for APOL3 (5). In silico structural folding of the MAD and C-terminal helices was determined by I-TASSER modeling (https://zhanggroup.org/I-TASSER/). The boxes illustrate the folding of the APOL1 or APOL2 N-terminal domain, as determined by X-ray scattering and nuclear magnetic resonance (4). In the isolated N-terminal domain, helix 5 can adopt two positions, preventing (bound) or not (open) helix 4 (or LZ1) accessibility. When accessible, like probably occurs in the full protein, helix 4 (or LZ1) can strongly interact with LZ2. Such LZ1-LZ2 pairing can promote cis-interaction between the HC1-LZ1 and HC2-LZ2 regions (6). This interaction is affected either by acidic conditions, as in trypanosome endosomes, or by LZ1 or LZ2 mutations (3). C-terminal mutations termed G1 and G2, which target LZ2, allow APOL1 to kill the APOL1-resistant human pathogen *T. brucei rhodesiense*, to the detriment of APOL1-mediated nephropathy (7).

Apolipoproteins-L (APOLs) are ubiquitous lipid-interacting proteins encoded by multigene families, whose rapid evolution suggests APOLs involvement in interactions with pathogens (1, 2). The human and mouse APOL families contain 6 and 12 members, respectively (murine APOLs are termed here mAPOLs). These proteins share a similar general structure, represented for APOL1 in Fig. 1. An N-terminal four helix-bundle precedes a double-stranded helix potentially capable of transmembrane insertion, followed by another double-stranded helix involved in membrane addressing and a C-terminal leucine zipper helix important for structural folding in the case of APOL1 (3) (Fig. 1). In addition, isoform-specific sequence insertion can occur, such as the N-terminal signal peptide addition that allows the secretion of a specific APOL1 isoform or sequence insertions in the N- terminal mAPOL7C region and at the C-terminal end of APOL6 and mAPOL6. In APOL1 and mAPOL6, these insertions confer specialized APOL functions, as discussed in specific sections.

APOLs are known since 1997, with the initial discovery of APOL1 as a component of bloodstream high-density lipoprotein particles (HDL-C) (8). This finding suggested a role of APOLs in lipid transport, particularly cholesterol, but the subsequent demonstration of APOL1 activity both in lysis of the African parasite *Trypanosoma brucei* (9) and in induction of kidney disease (7) suggested more complex functions. In dendritic cells (DCs) or in the DC-like kidney podocytes, the expression of APOL1, APOL3, and murine APOL3-like mAPOL7C is strongly induced by the viral mimetic poly(I:C), which triggers cellular signalling by type-I interferon (IFN-I) and involves the Toll-like receptor-3/TIR domain containing adapter-inducing interferon β pathway (6, 10), and in epithelial cells, APOL3 is induced by INF-γ (11). Thus, these APOLs can be considered as encoded by interferon-stimulated genes. Specific roles of these proteins have been identified in the membrane rearrangements linked to inflammation and induction of immunity (3, 12). Accordingly, membrane-targeting activities were also recently discovered for other APOL isoforms, such as APOL2, mAPOL8, and mAPOL9. Collectively, the evidence points to a basic role of APOLs in the control of membrane dynamics, particularly under inflammatory conditions.

### Possible APOLs’ roles in membrane dynamics for STING activation and degradation

Activation of the pathogen DNA-sensor Stimulator of interferon genes (STING), a transmembrane protein of the endoplasmic reticulum (ER), depends on its translocation from the ER to the Golgi for oligomerization at the Golgi surface, which induces inflammatory gene expression and IFN-I signalling (13). Detection of pathogen DNA triggers the synthesis of cyclic GMP-AMP, which binds to STING and induces transient cholesterol esterification, lowering free cholesterol levels, disrupting STING–cholesterol interactions at the ER, and allowing STING translocation to the Golgi (14). This translocation crucially depends on STING interaction with phosphatidylinositol-4-phosphate (PI4P), a phospholipid specifically synthesized by PI4-kinase-B (PI4KB) at the Golgi (15, 16, 17). PI4P is involved in a transmembrane helix rearrangement that promotes STING oligomerization and consecutive activation (18). PI4P is also known to recruit Golgi-phosphoprotein-3 (GOLPH3), a Golgi membrane curving agent essential for vesicular fission and traffic, notably through association with ADP-ribosylation-factor-1 (ARF1) and indirect interaction with nonmuscular-myosin-2A (NM2A) (19, 20). GOLPH3 could facilitate the association of curved STING oligomers (21) with Golgi vesicles. Accordingly, the GOLPH3-like protein, a PI4P-binding protein that antagonizes the action of GOLPH3 (22), can interact with STING to promote its retrograde transport from the Golgi to the ER (23).

Like STING, APOL1 is also an ER-associated protein able to interact with both cholesterol and PI4P (3, 6, 12). Thus, the transient reduction of ER cholesterol due to infection could allow not only STING but also APOL1 traffic from the ER to the Golgi. Accordingly, cellular treatment with poly(I:C) increases the Golgi/ER ratio of APOL1, contrasting with a Golgi/ER APOL3 ratio decrease by the same treatment (6).

**Figure 2.**
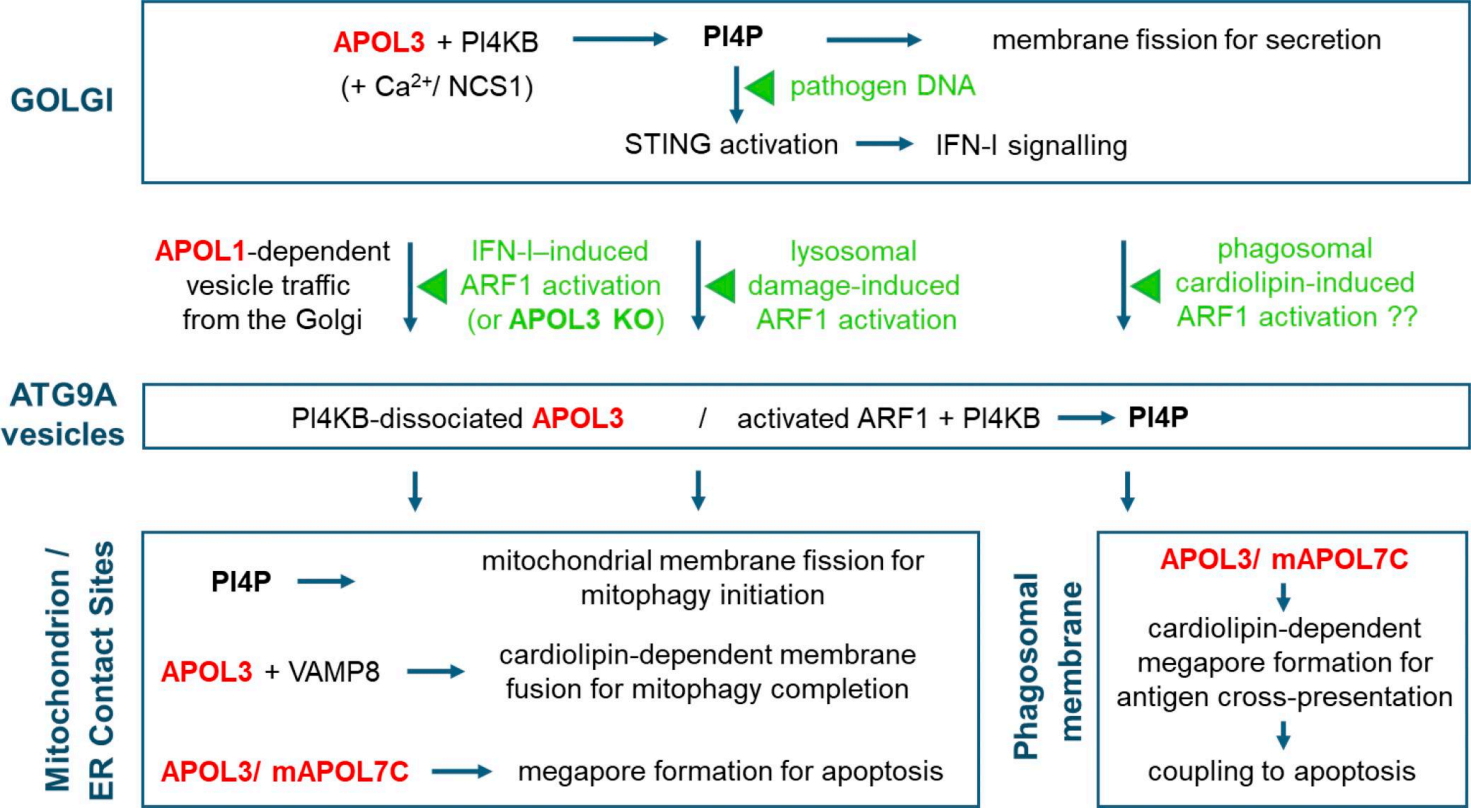
**APOL1 and APOL3 involvement in inflammation and antigen cross-presentation.** The cellular detection of pathogen DNA triggers activation, then degradation, of the inflammation stimulator STING. This occurs through PI4P-mediated translocation of STING from the ER to the Golgi, followed by STING oligomerization at the Golgi, then STING translocation from the Golgi to MERCS (13). APOL3 could be involved in this process through its key role in promotion of PI4P synthesis by the PI4KB kinase at the Golgi. Either IFN-I signalling or APOL3 downregulation (loss or inactivation) triggers the traffic of Golgi-derived ATG9A vesicles carrying PI4KB and ARF1 to MERCS. STING also traffics in such vesicles for its degradation, but whether this involves APOL1 and/or APOL3 is not known. If activated by IFN-I, ARF1 is expected to dissociate APOL3 from PI4KB (24), allowing PI4KB release from the Golgi. ARF1-induced PI4KB activity at MERCS initiates mitophagy through mitochondrion fission. If trafficked to MERCS following APOL3 downregulation, thus, in the absence of IFN-I signalling, inactivated ARF1 may not allow efficient induction of mitophagy, causing podocyte dysfunctions such as occurs in APOL1-mediated nephropathy (24). Mitophagy completion results from mitophagosome fusion with endolysosomes, which involves APOL3 interaction with the endosomal fusion protein VAMP8 (24). Thus, APOL3 and STING are markers of both initiation and termination of inflammation. Cardiolipin insertion in the DC phagosomal membrane, occurring during phagocytosis of pathogens, could hypothetically trigger APOL3- or mAPOL7C-driven megapore formation, allowing antigen cross-presentation. Accordingly, APOL3 and mAPOL7C are also involved in apoptotic megapore formation in cardiolipin-containing mitochondrial outer membrane, either following IFN-I signalling or lysosomal damage. In both cases, APOL3 may interact with VAMP8, known as APOL3 to be involved in both mitophagy and antigen cross-presentation.

APOL3 exhibits specific interactions with different proteins involved in PI4P synthesis at the Golgi, such as PI4KB, ARF1, and the PI4KB-controlling factors neuronal calcium sensor-1 (NCS1) and calneuron-1, and in the presence of calcium, APOL3 can directly promote PI4KB activity (6, 24). At the Golgi, this activity is involved in vesicle secretion (19) (Fig. 2). In the absence or inactivation of APOL3, PI4P synthesis at the Golgi is severely reduced, linked to extensive Golgi shrinkage and Golgi-derived traffic of vesicles containing the lipid scramblase autophagy-9A (ATG9A), which carry PI4KB and ARF1 out of the Golgi (6, 24, 25). Given the crucial role of APOL3 in Golgi PI4P synthesis by PI4KB and the key importance of PI4KB-synthesized PI4P for STING oligomerization at the Golgi, APOL3 is likely to be involved in STING activation. So far, whether APOL3 can interact with STING and/or GOLPH3 is unknown, but all three proteins share both binding to PI4P and association with NM2A (6, 20, 26).

The induction of IFN-I signalling triggers ARF1 activation (GDP to GTP association), together with PI4KB, ARF1, APOL1, and APOL3 trafficking in Golgi-derived ATG9A vesicles to mitochondrion-ER contact sites (MERCS), for induction and completion of auto/mitophagy and apoptosis (24) (Fig. 2). More than 90% of cytoplasmic APOL1 is associated with ATG9A vesicles, and APOL1 is required for the traffic of these vesicles from the Golgi to MERCS, where mitophagy is induced (24). Such activity probably involves the demonstrated APOL1 interactions with the NM2A myosin and the mitophagy receptor prohibitin-2 (PHB2) (6, 24). Interestingly, NM2A-driven traffic of ATG9A vesicles from the Golgi to MERCS is also involved in the induction of STING degradation and inflammation termination (13, 27, 28, 29, 30, 31, 32). Thus, STING could be associated with APOL1 and APOL3 in ATG9A vesicles.

As an alternative to IFN-I signalling, the loss or inactivation of APOL3 also triggers Golgi-derived ATG9A vesicle traffic to MERCS (24). Thus, APOL3 controls ARF1/PI4KB sequestration at the Golgi under noninflammatory conditions, and APOL3 downregulation could possibly affect not only ARF1/PI4KB but also STING trafficking. Whereas auto/mitophagy initiation involves the membrane fission activity of ARF1/PI4KB (25, 33, 34), auto/mitophagy completion involves the membrane fusion activity of both APOL3 and STING (24, 32). Interestingly, STING can interact with the autophagic fusion protein syntaxin-17 (STX17) (32), whose function precisely appears to be played by APOL3 in the case of mitophagy (24).

In conclusion, APOL3 and APOL1, respectively, control Golgi PI4P synthesis by PI4KB and Golgi-derived ATG9A vesicle trafficking, which also underlies STING activation for inflammation induction and STING degradation for inflammation termination. Therefore, APOL1 and APOL3 may be involved in STING activation and degradation. Interestingly, in HeLa cells, the absence of APOL3 prevented STING activation induced by lysosomal damage (35, *Preprint*). Although this was ascribed to the absence of mitochondrial DNA leakage, inability to trigger PI4P-induced STING activation by PI4KB cannot be ruled out.

### APOLs’ roles in mitochondrial membrane dynamics for inflammatory damage repair

Either activation of IFN-I signalling or APOL3 downregulation (APOL3 absence or APOL3 inactivation) leads to Golgi shrinkage, together with Golgi-derived trafficking of PI4KB-carrying ATG9A vesicles to MERCS (6, 24) (Fig. 2). In case of IFN-I, this traffic could be induced by ARF1 activation because ARF1 activation is expected to release PI4KB from APOL3 interaction, mimicking the stimulatory effect of APOL3 loss on PI4KB traffic induction (24). Interestingly, lysosomal damage can also induce APOL3 translocation to the mitochondrion (35, *Preprint*). Since lysosomal damage induces the activation of ARF1 (36), ARF1 activation is possibly involved in APOL3 traffic to the mitochondrion following either pathogen infection or lysosomal damage.

The Golgi-derived ATG9A vesicle traffic requires APOL1, as it does not occur in APOL1 absence (24). Accordingly, APOL1 is tightly associated with the NM2A myosin (6), known to drive ATG9A trafficking (28). This traffic is probably directed to the mitochondrion by the mitophagy receptor PHB2, which binds to both APOL1 (24) and GOLPH3 (37). The NM2A-driven ATG9A vesicles carry the fission factors PI4KB, ARF1, and GOLPH3, transferring this membrane fission machinery to the mitochondrion (25, 34, 38, 39). APOL1 involvement in this traffic provides an explanation for the inhibition of mitochondrial membrane fission in APOL1 KO podocytes (24). PI4P-enriched microdomains at the mitochondrial surface may allow the recruitment of fission-1, which is involved in stress-induced fission and mitophagy (3, 40).

Mitochondrion fission generates mitophagosomes that undergo fusion with endolysosomes for mitophagy completion, a crucial repair mechanism removing dysfunctional mitochondria damaged by inflammation (41). The fusion of mitophagosomes with endolysosomes requires the interaction of APOL3 with the endosomal fusion protein vesicle-associated-membrane-protein-8 (VAMP8) (24). Such APOL3 involvement in membrane fusion provides an explanation for the accumulation of mitophagosomes in APOL3 KO podocytes (24). Whether STING also participates in membrane fusion completing mitophagy is unknown, but STING interacts with both fusion factors STX17 and VAMP8 (32), and VAMP8 interacts with both APOL3 and STX17 (24).

In conclusion, through their key involvement in induction and completion of mitophagy, APOL1 and APOL3 contribute to avoiding detrimental effects of infection-induced inflammation on the mitochondrial function. Consistently, interference with APOL1 or APOL3 activities induces severe auto/mitophagy dysfunctions, causing impairment of mitochondrial activity (24), as it occurs in APOL1-mediated nephropathy (3, 12).

### APOLs’ roles in membrane permeabilization for antigen cross-presentation and apoptosis

Cross-presentation of phagocytosed antigens at the surface of DCs triggers T cell responses against infection. This process prevents antigen digestion in the lysosome through phagosomal membrane permeabilization, allowing antigen release in the cytoplasm before membrane fusion occurs between the phagosome and endolysosomes. In mice, antigen cross-presentation requires the APOL3-like mAPOL7C (42), which is strongly induced in DCs by IFN-I-mediated inflammation (10). Accordingly, human APOL3 can also trigger phagosomal permeabilization (42), and the APOL3 membrane permeabilization activity appears to promote T cell activation (35, *Preprint*, 43). This activity is in keeping with the membrane fusion activity of APOL3 during mitophagy (24). However, in contrast to APOL3, mAPOL7C contains a specific acidic stretch insertion disrupting the N-terminal domain, which could affect membrane fusion activity (3).

To allow antigen escape from the phagosome, the phagosomal membrane should be permeabilized by megapores distinct from ion pores. I propose that membrane permeabilization by mAPOL7C/APOL3 results from megapore formation triggered by cardiolipin membrane insertion during phagocytosis. Due to the absence of cardiolipin in the phagosomal membrane, mAPOL7C/APOL3 fusion activity is expected to be low under normal conditions. Indeed, APOL3 plays a specific role in cardiolipin solubilization (35, *Preprint*), and this activity likely accounts for the important contribution of cardiolipin in APOL3-driven membrane fusion assays (24). Insertion of cardiolipin in the phagosomal membrane, which occurs during phagocytosis of cardiolipin-exposing membranes from pathogens (44), could induce damage-associated molecular pattern signalling (45), triggering mAPOL7C/APOL3 recruitment from the Golgi to the phagosomal membrane. Cardiolipin would stimulate mAPOL7C/APOL3 membrane fusion activity, causing phagosomal megapore formation at cardiolipin-containing sites. Consistently, VAMP8, the APOL3 partner for membrane fusion, is present at the phagosomal membrane during phagocytosis, where it is known to be crucially involved in antigen cross-presentation (46).

mAPOL7C/APOL3 activity is also generally linked to apoptosis, which involves megapore formation in cardiolipin-exposing mitochondrial outer membrane. Indeed, DCs are programmed for apoptosis, presumably to limit inflammation (47) but also to promote antigen cross-presentation (48, 49). Moreover, like APOL1 and APOL3 in human podocytes, mAPOL7 and mAPOL11 isoforms are clearly involved in murine DCs apoptosis induced by poly(I:C) because downregulation of these APOLs strongly inhibits apoptosis (6, 10). Furthermore, expression of the mAPOL7 and mAPOL11 isoform families specifically occurs in the CD8α+ subset of DCs (10), which are characterized by their antigen cross-presentation ability, together with a shorter lifespan than other DCs (50). Finally, mAPOL7 isoforms can associate with the anti-apoptotic protein B-cell-lymphoma-extra-large (Bcl-xL), suggesting a potential role for relieving anti-apoptotic activity (10). Accordingly, inhibition or deletion of the Bcl-xL-related anti-apoptotic B-cell-lymphoma-2 (BCL2) protein triggers enhanced DC antigen presentation, suggesting a possible role of pro-apoptotic BCL2 family members in antigen cross-presentation (51).

The coupling between mAPOL7C-/APOL3-mediated phagolysosomal membrane permeabilization and apoptosis strikingly evokes the APOL1- or APOL3-mediated coupling of lysosomal and mitochondrial membrane permeabilization in trypanosomes (5, 52), as well as the coupling between lysosomal damage and mitochondrial DNA efflux induced by APOL3 in HeLa cells (35, *Preprint*). In trypanosomes, this coupling involves endolysosomal membrane traffic by the cholesterol-trafficking TbKIFC1 kinesin (52, 53). In DCs, it could result from activated ARF1-driven transfer of APOL3-carrying ATG9A vesicles from the Golgi to both phagolysosomal and mitochondrial membranes. Indeed, in addition to its traffic to MERCS for mitophagy completion (Fig. 2), APOL3 could also be trafficked to phagolysosomal membranes because ATG9A vesicle trafficking is not only involved in mitophagy but also in phagocytosis (54), and Golgi-derived traffic of ARF1-associated vesicles is known to be involved in phagocytosis (55). Similarly, in HeLa cells, the coupling between lysosomal damage and APOL3-mediated mitochondrial membrane permeabilization could involve activated ARF1-mediated traffic of APOL3 from the Golgi (35, *Preprint*, 36). Interestingly, in this case as for mitophagy induction at the mitochondrion (Fig. 2), ATG9A vesicle trafficking promotes delocalized PI4P synthesis (56). However, this traffic, which induces the repair of damaged lysosomal membranes, involves the endolysosomal-specific PI4K2A kinase instead of PI4KB. So far, PI4K2A is not known to be controlled by APOL3, and it is not required for STING activation and traffic.

The mechanism for APOL3/APOL7C-mediated megapore formation remains to be clarified. So far, factors known to be involved together with APOL3/mAPOL7C in cross-presentation-linked membrane permeabilization include the endosomal sorting complex required for transport III (42), which functions with NM2A (an APOL1-associated myosin) for endolysosomal membrane fission (57), and the fusion protein VAMP8 (46), a necessary APOL3 partner for mitochondrial membrane fusion with endolysosomes during mitophagy (24). Thus, it may be envisaged that megapores are formed during membrane fusion between cardiolipin/APOL3-exposing phagosomal membranes and VAMP8-exposing endolysosomal membranes. Since promoting autophagy also promotes antigen cross-presentation (58), these processes may involve similar membrane interactions. Given both the interaction of mAPOL7 isoforms with the anti-apoptotic BCL2-like protein Bcl-xL (10) and the BCL2 reducing activity for antigen presentation by DCs (51), pro-apoptotic BLC2 family members like BAX or BAK could be involved in cross-presentation-linked megapore formation. Finally, given both its synergistic activity with APOL3 in bacterial membrane permeabilization (11) and its common interaction properties with APOL3 (see paragraphs below on bacteria and parasite control), guanylate-binding protein-1 (GBP1) is likely to participate with APOL3/mAPOL7C in antigen cross-presentation by inducing the cytosolic release of molecules from various intracellular pathogens.

In conclusion, antigen cross-presentation by DCs requires membrane megapore formation by mAPOL7C or APOL3, generally coupled to apoptosis induction. Such activity is likely to depend on cardiolipin exposure in the phagosomal membrane, together with cardiolipin-dependent APOL3 fusion activity. Through their activity in both inflammation and antigen presentation, APOLs appear to influence both innate and adaptive immunity.

### APOLs’ roles in vesicle exocytosis for liver fibrosis and gut mucosal immunity

Two putative murine APOL2 homologs, namely mAPOL8 and mAPOL9, have recently been involved in vesicle exocytosis and membrane fusion activity. APOL2 or mAPOL8 were found to be crucially involved in liver fibrosis, a transforming-growth-factor-β1 (TGF-β)-induced disease linked to exocytotic trafficking of profibrogenic vesicles (59). In hepatic stellate cells, this activity requires APOL2/mAPOL8 interaction with a lipid mimicked by the hydrophobic drug 12-deoxyphorbol-13-palmitate (59). Similarly, mAPOL9 was found to be involved in the induction of immunogenic outer membrane vesicle formation at the surface of gut bacteria, through interaction with ceramide-1-phosphate at the bacterial membrane (60).

**Figure 3.**
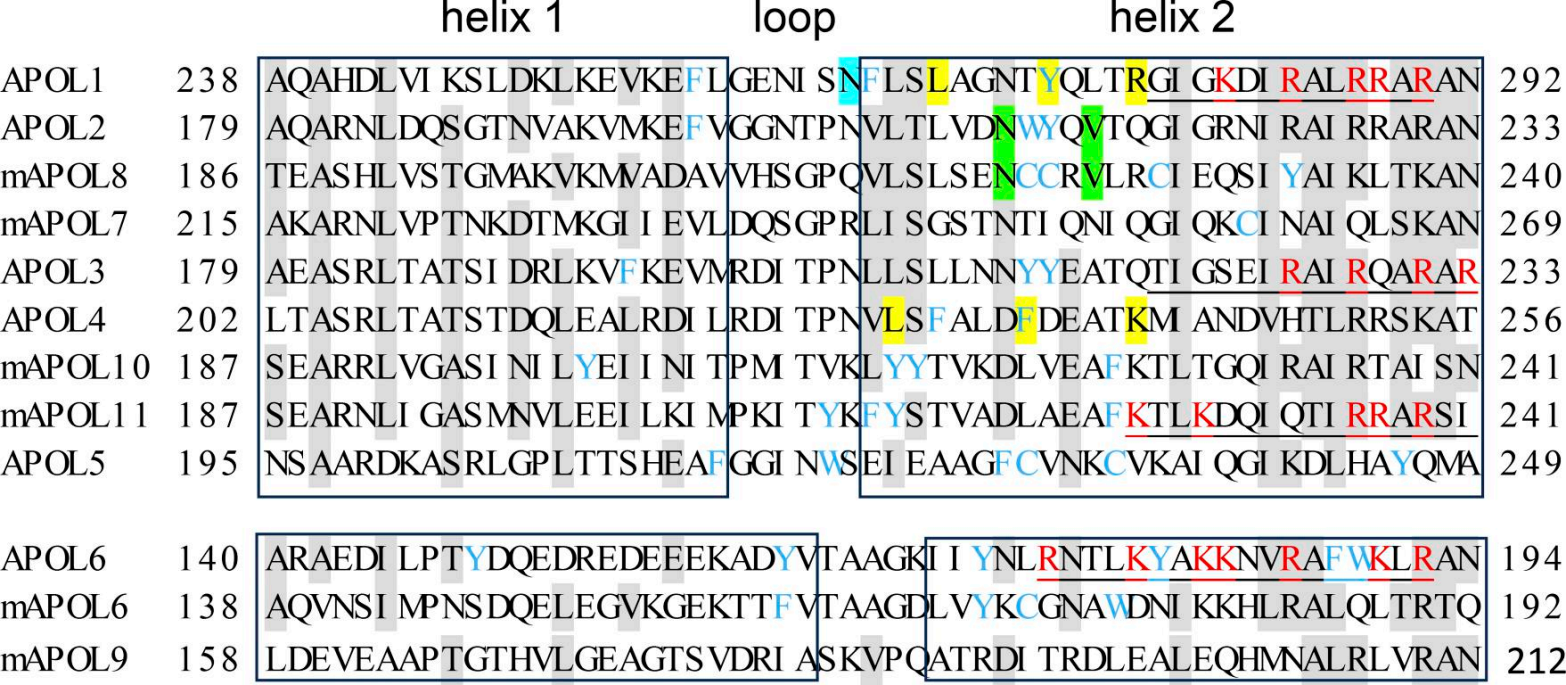
**The APOL membrane-addressing domain.** Identical or similar amino acids shared between most APOLs are highlighted in grey. Despite sequence divergence with other MAD isoforms, the (m)APOL6 and mAPOL9 MADs can also be structured in a double-stranded hairpin helix. Probable calmodulin-binding sites (http://calcium.uhnres.utoronto.ca/ctdb/ctdb/sequence.html), which also interact with phosphoinositides or cardiolipin (67, 68, 69), are underlined. Positively charged amino acids potentially involved in phospholipid interactions are colored red. In APOL1, N264, highlighted in blue, is essential for the cytotoxic activity of C-terminal APOL1 variants (65, 66). The amino acids highlighted in yellow define a consensus sequence for interaction with cholesterol (62). The amino acids highlighted in green are essential for interaction with the antifibrotic drug 12-deoxyphorbol-13-palmitate (59). Of note, the MAD domain is rich in cysteines (C) and/or aromatic residues (F, Y, and W), especially in the first half of the second helix (colored light blue). MAD, membrane-addressing domain.

Lipid interaction with APOL2 or mAPOL8 involves a discrete motif in the second helix of the double-stranded hairpin helix in the APOL2/mAPOL8 membrane-addressing domain (MAD) (59, 61) (Fig. 3). Strikingly, the key lipid-interacting amino acids of APOL2 and mAPOL8 are located precisely in a region corresponding to that allowing interaction of APOL1 with cholesterol (cholesterol recognition amino acid consensus, or CRAC) (61, 62, 63). Similarly, a putative CRAC sequence in the same region of APOL4 (Fig. 3) may condition APOL4 cholesterol transport activity (64). Moreover, this region is close to an amino acid whose mutation (N264K) induces a strong reduction of C-terminal APOL1 variants cytotoxicity acting on the kidney podocyte plasma membrane (65, 66), suggesting a key involvement in membrane targeting. Accordingly, this region is flanked by stretches alternating positively charged and hydrophobic amino acids, as expected for phospholipid-binding peptides. More precisely, MAD helix 2 of APOL1, APOL3, APOL6, and mAPOL11 contains a probable calmodulin-binding site, which is also expected to interact with anionic phospholipids such as phosphoinositides or cardiolipin (67, 68, 69). Thus, the second helix of the membrane-addressing domain appears to be crucial for membrane interaction that underlies the activity of different APOLs. Interestingly, this domain is rich in cysteines and/or aromatic amino acids, especially in the first half of the second helix (Fig. 3). Such amino acids are likely to participate in surface interactions.

Given the absence of a signal peptide in APOL2, mAPOL8, or mAPOL9, the extracellular release of these proteins is likely to result from exocytosis of profibrogenic APOL2-or mAPOL8/mAPOL9-coated vesicles (61). Accordingly, TGF-β1 is known to promote APOL2 expression, extracellular vesicle secretion, and gut immunity (59, 70). Since both bacterial outer membrane budding and liver fibrosis involve vesicular membrane fusion (60, 71), APOL2, mAPOL8, and mAPOL9 appear to be endowed with membrane fusion activity, implying a transmembrane association.

In conclusion, APOL2 or mAPOL8/mAPOL9 induction by TGF-β participates in exocytotic vesicle trafficking, causing liver fibrosis or controlling gut mucosal immunity, possibly through extracellular vesicular membrane fusion dependent on specific lipid recognition.

### APOLs’ roles in resistance to intracellular bacteria

As stated above, APOL3 can induce mitochondrial membrane fusion both in kidney podocytes and in trypanosomes, owing to its double-stranded hairpin helix able of pH-independent transmembrane insertion, which contrasts with the acidic pH dependence of this activity in the case of APOL1 (5, 24). This transmembrane hairpin helix is characterized by its high flexibility (high proportion of β-branched [Ile/Val] and helix-breaking [Gly/Pro/Ser/Thr] amino acids), a key feature for membrane fusion proteins (72), and it is flanked on both sides by long α-helices able to interact both with fusion factors like VAMP8 (24) and with anionic phospholipids, like PI4P or cardiolipin (Fig. 3). In epithelial cells, such characteristics possibly account for the detergent-like effect of APOL3 on membranes of the intracellular bacteria *Salmonella typhimurium*, which involves the formation of vesicular-like structures with bacterial membrane debris (11). Accordingly, cardiolipin, a characteristic lipid of mitochondrial and bacterial membranes, was found to interact with both APOL3 and VAMP8 and to strongly stimulate in vitro APOL3 fusion activity (24), probably owing to the specific APOL3 ability to solubilize cardiolipin (35, *Preprint*). Thus, APOL3-mediated solubilization of cardiolipin may be coupled to a similar activity by VAMP8. Moreover, digestion of bacterial residual components is expected to involve endolysosomal VAMP8 like it occurs during mitophagy. However, there was no requirement for VAMP8 in *S. typhimurium* restriction within IFN-γ-activated human cells (11).

**Figure 4.**
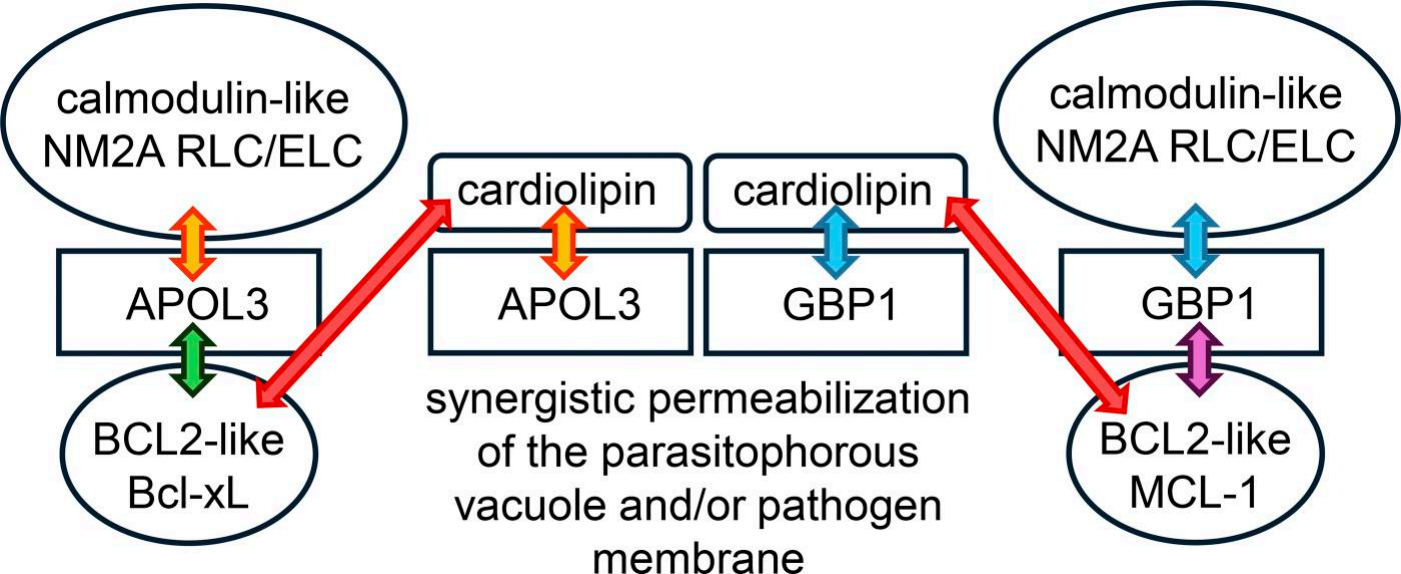
**Synergistic APOL3 and GBP1 activities.** Both proteins participate in the permeabilization of intracellular bacterial membranes (11). Given their common interaction properties (double arrowheads), they could hypothetically also participate in permeabilization of various pathogen-surrounding phagosomal membranes (73, 74, 75), promoting both inflammation and antigen cross-presentation. Of note, cardiolipin and calmodulin share the same binding sites, including in the myosin heavy chain (69).

One synergistic APOL3 partner appears to be the IFN-γ-induced GBP1 (11), a PI4P-binding protein involved in phagosomal/endolysosomal membrane repair (73). This protein directly acts with APOL3 to reduce bacterial viability (11, 74), likely through its dynamin-like GTPase activity for membrane permeabilization (75). Interestingly, mouse GBP2, the ortholog of human GBP1, exhibits high affinity for cardiolipin (76), suggesting a similar activity for GBP1 (probable cardiolipin-binding helix: G432-E449). In addition, like APOL1 or APOL3, GBP1 associates with NM2A myosin regulatory light chain (RLC) and essential light chain (ELC) (6, 75), which are both calmodulin-like proteins. Thus, GBP1 and APOL3 may collaborate for membrane permeabilization that involves both GTPase and NM2A activities (Fig. 4). Interestingly, while the APOL3-like mAPOL7 isoforms interact in DCs with the anti-apoptotic BCL2-like protein Bcl-xL (10), GBP1 interacts with the macrophage BCL2-like protein MCL-1 (myeloid cell leukemia-1) (77), suggesting the involvement of both APOL3 and GBP1 in apoptotic-like membrane megapore formation. Like APOL3 and GBP1, BCL2, Bcl-xL and MCL-1 all contain a calmodulin-binding site allowing cardiolipin interaction.

In conclusion, the bactericidal activity of APOL3 may involve the same membrane fusion activity as that exhibited for the completion of mitophagy, with a specific participation in cardiolipin solubilization. Since so far no APOL3 mutations have been associated with immunodeficiency, it is currently unknown to which extent APOL3 contributes to antibacterial immunity. The APOL3 role in human susceptibility or resistance to *Salmonella*, as well as the possibility of APOL3 targeting other intracellular bacterial pathogens, remains to be clarified. Given the known association of GBPs with intracellular membranes surrounding *Shigella flexneri*, *Burkholderia thailandensis*, *Legionella pneumophila*, *Yersinia pseudotuberculosis*, *Brucella abortus*, *Chlamydia trachomatis*, *Mycobacterium tuberculosis,* and *Listeria monocytogenes* (73), GBPs and APOL3 could also be involved in the control of these bacterial pathogens. However, despite its role in antibacterial activity, APOL3 is currently not associated with inborn errors of immunity (IEIs).

### APOLs’ roles in viral infection control

Inflammatory signalling induced by virus detection strongly increases APOL1, APOL3, mAPOL7, mAPOL9, and mAPOL11 expression (6, 10, 78, 79). Consistently, APOLs are involved in either anti- or proviral activities, depending on the virus type (79, 80, 81, 82, 83). Whereas APOL1 restricts HIV-I infection (80), mAPOL9 inhibits replication of Theiler’s murine encephalomyelitis virus but not replication of either vesicular stomatitis RNA virus or Murid herpesvirus-4 DNA virus (79), and conversely, APOL3 behaves as a proviral factor for flavivirus replication (83).

Among other possibilities linked to specific viral infection strategies, such contrasting pattern may result from the fact that PI4KB-synthesized PI4P is not only an activator of STING signalling, which operates against infection by DNA viruses, but it is also a key building material for the construction of virus replication platforms, explaining why different viruses hijack PI4KB (84) or STING (85). Thus, APOL1 and mAPOL9 may restrict viral infection through their common association with the mitophagy receptor PHB2, which could prevent PI4KB proviral activity by directing the PI4KB traffic to the mitochondrion (24). Conversely, in other cases, the same activity may promote proviral building of the virus replication platform, depending on the virus replication strategy (84).

While APOLs involvement in infection by DNA viruses may relate to PI4P synthesis, the activity of the Mitochondrial Antiviral Signalling MAVS protein, which is specifically involved in the response to RNA viruses (86), could also be controlled by APOLs. Indeed, mitophagy is critical for the degradation of MAVS (87), and APOL3 is essential for completion of mitophagy (24). Thus, APOL3 may exhibit proviral activity through MAVS down-regulation. Accordingly, following IFN-I-mediated promotion of mitophagy, APOL3 is a potent proviral factor promoting the replication of different flaviviruses, as well as several other related and unrelated RNA viruses, and this activity is independent from PI4KB (83).

In conclusion, APOLs could affect viral infection differently depending on the nature of the virus genome and replication strategy. Whereas the PI4KB-controlling activities of APOL1 and APOL3 may influence the replication of DNA viruses, APOL3 mitochondrial membrane fusion activity may promote infection by RNA viruses independently of PI4KB, through the control of mitophagy completion.

### APOLs’ roles in parasite infection control and linkage with kidney disease

The first function attributed to APOLs was APOL1 activity as innate immunity factor against the bloodstream protozoan parasite *T. brucei brucei*, responsible for sleeping sickness in Africa (9). This activity has been largely detailed (88, 89). In brief, trypanosome lysis is due to a secreted isoform of APOL1, circulating in the blood through association with HDL-C particles. These particles also carry haptoglobin-related protein (HPR), a protein able to interact with a specific trypanosome surface receptor, which allows efficient APOL1 uptake in parasite endosomes (90). Trypanosome lysis results from apoptotic-like mitochondrial membrane permeabilization induced by acidic pH-dependent APOL1 insertion into endosomal membranes, followed by intracellular trafficking of these membranes to the mitochondrion (52). Moreover, in keeping with its fusion-promoting ability after transmembrane insertion (24), APOL1 also promotes the fusion of trypanosome mitochondrial membranes (52), likely involving APOL1 interaction with the trypanosome VAMP8 homolog TbVAMP7B (24). Interestingly, a helix 5 sequence resembling the BCL-2 homology 3 “death” domain of apoptotic BCL2 family proteins (156-RRLRALADGV-164) is crucial for APOL1-mediated megapore formation in trypanosome mitochondrial membranes, although it is not required for APOL1 association with mitochondria (52). Given the apparent absence of BCL2 family proteins in trypanosomes, this sequence is unlikely to interact with apoptotic proteins, but could rather be involved in cardiolipin interaction. Indeed, APOL1 or APOL3 helix 5 contains a probable calmodulin-binding site, and such sites are also known to interact with anionic phospholipids like cardiolipin (67, 68, 69). How APOL1 induces megapore formation in the trypanosome mitochondrion is still unclear.

**Figure 5.**
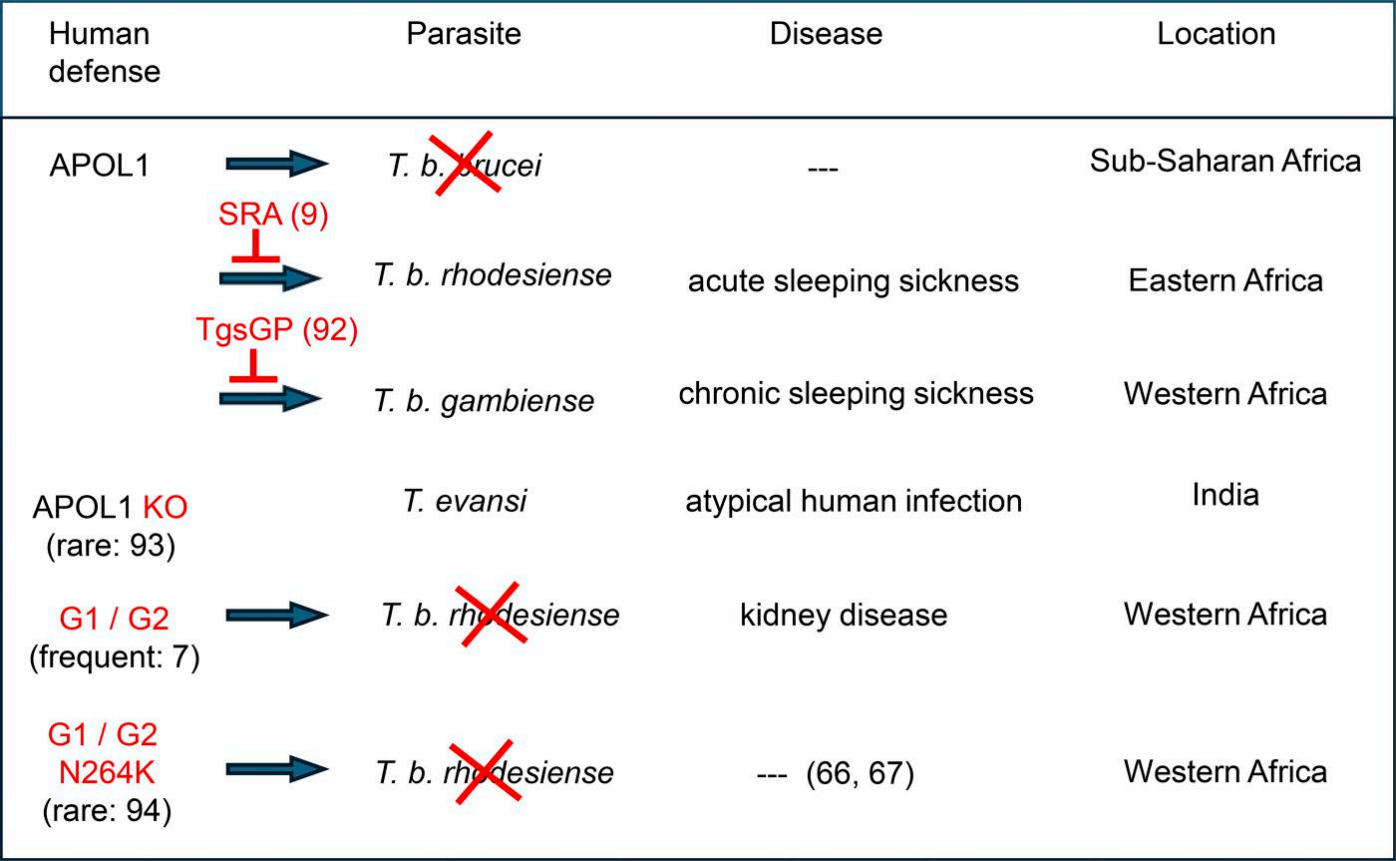
**Molecular ‘arms race’ between African trypanosomes and humans.** Human ancestors produced a secreted version of APOL1 to kill the bloodstream parasite *T. brucei brucei* (9). Two *T. brucei brucei* clones, known as *T. brucei rhodesiense* and *T. brucei gambiense*, can neutralize APOL1 through either direct interaction of the *T. brucei rhodesiense*-specific SRA with APOL1, or *T. brucei gambiense*-specific TgsGP interference with APOL1 activity (9, 91). A rare Indian case of APOL1 KO due to frameshift mutations in both APOL1 alleles resulted in atypical infection by *T. brucei* brucei-like *T. evansi* parasites (92). The APOL1 C-terminal variants G1 and G2, widespread in Western Africa, can restore human resistance to *T. brucei rhodesiense* through interference with SRA–APOL1 interaction (7). However, the G1 or G2 mutations also confer high probability of humans developing chronic kidney disease (7). APOL1 variants with the N264K mutation can still efficiently kill *T. brucei rhodesiense* but no longer exhibit kidney podocyte cytotoxicity (65, 66, 93) ( →: APOL1 trypanolytic activity; ✖: trypanosome lysis; ---: no disease).

Remarkably, the extracellular function of APOL1 as a lytic factor for bloodstream parasites is performed through the exclusive appearance of secretion among all known APOL isoforms, only occurring in some primates, including humans, and thus recently in evolution. Extracellular activities of other isoforms such as APOL2, mAPOL8, or mAPOL9 (59, 60, 61) presumably occur through association with exocytotic vesicles. This originality of APOL1 is likely due to the imperative necessity for primates to resist the local bloodstream pathogen *T. brucei brucei* for their development in Africa. Consistently, following the generation of the APOL1-resistant parasite *T. brucei rhodesiense*, which can neutralize APOL1 through direct interaction with a *rhodesiense*-specific protein termed serum resistance-associated (SRA) (9), C-terminal APOL1 variants readily appeared in Western Africa to restore APOL1 ability to kill this resistant parasite. These variants, termed G1 and G2, respectively. contain the double S342G/I384M mutation and the double N388/Y389 deletion, which disrupt the APOL1 inhibitory interaction of SRA (7). Thus, the G1 and G2 variants specifically protect humans against *T. brucei rhodesiense* infection. Such protection probably explains why these APOL1 variants are widespread in Western Africa, probably leading to *T. brucei rhodesiense* disappearance from this region. Instead, another APOL1-resistant trypanosome termed *T. brucei gambiense* is present in Western Africa, causing chronic sleeping sickness. This parasite is largely resistant to either WT or G1/G2 APOL1 through *T. brucei gambiense*-specific glycoprotein (TgsGP)-mediated prevention of APOL1 insertion in endosomal membranes (91). It is probable that *T. brucei rhodesiense* disappearance allowed *T. brucei gambiense* to propagate (88) (Fig. 5).

APOL1 C-terminal G1 and G2 variants that allow lysis of APOL1-resistant trypanosomes are also responsible for a disease, namely APOL1-mediated nephropathy (7). This disease includes focal segmental glomerulosclerosis, hypertension attributed end-stage kidney disease, collapsing glomerulopathy, and sickle cell nephropathy. The G1/G2-linked pathology involves the detachment of podocytes from glomeruli following podocyte foot process effacement, pointing to major rearrangements of the actomyosin cytoskeleton. Under noninflammatory conditions, such actomyosin rearrangements induce reduced auto/mitophagy flux and mitochondrial dysfunctions, all attributable to APOL3 inactivation by the APOL1 variants (6, 24, 63). Accordingly, either natural mutations preventing APOL3 expression, or experimental APOL3 deletion (APOL3 KO), cause a similar phenotype (6, 24, 94, 95). Considering the possible roles of APOL1 and APOL3 in STING activation and degradation, the hypothesis that kidney disease results from podocyte dysfunctions linked to APOL1-mediated APOL3 inactivation also accounts for the involvement of STING in these dysfunctions (96).

Under inflammatory conditions like viral infection, APOL1-mediated nephropathy exhibits a more severe pathology, due to specific cytotoxicity exerted by G1 or G2 APOL1 on the podocyte plasma membrane (97). This toxicity correlates with the amount of secreted APOL1 in the kidney (97). Accordingly, signal peptide-mediated ER translocation, which leads to extracellular secretion, is absolutely required for APOL1 risk variant cytotoxicity, and intracellular APOL1 risk variant isoforms are harmless (98, 99). Moreover, the pattern of APOL1 epitopes accessible to monoclonal antibodies at the podocyte surface is better explained by surface binding of extracellular (secreted) APOL1, rather than by transmembrane insertion (100). This cytotoxicity results from stress induced by increased cationic fluxes at the cell surface (101, 102, 103). I have proposed that such effects result from inflammation-linked increase of G1 or G2 variant interaction with cholesterol at the podocyte membrane, activating the cholesterol-controlled cation channels transient receptor potential channel-6 (TRPC6) and BK (big K) channel, which, respectively, induce Ca^2+^ and Ca^2+^-dependent K^+^ plasma membrane fluxes crucially involved in podocyte function (63). On one hand, inflammation induces both enhanced expression of APOL1 and enhanced cholesterol-binding potential of secreted APOL1 within the kidney, due to inflammation-mediated amount lowering of APOL1-sequestering and APOL1-circulating HDL-C particles. On the other hand, G1- or G2-mediated disruption of cis-interaction between the LZ2 and LZ1 helices enhances APOL1 hydrophobicity, increasing APOL1 binding to cholesterol (3, 6, 63, 104). Thus, these combined effects result in higher APOL1 variants interaction with podocyte cholesterol under inflammatory conditions. Accordingly, the N264K mutation, which is expected to affect APOL1 binding to cholesterol (63), prevents APOL1 G2 cytotoxicity (65, 66). In support to this model, APOL1 variants induce increased APOL1 clustering in membrane microdomains (99). Besides the well-known involvement of the TRPC6 and BK channels in kidney disease, their coupled activation by cholesterol provides a straightforward interpretation for the coupled increase of Ca^2+^ and K^+^ fluxes underlying podocyte dysfunction by the APOL1 variants (63).

As an alternative explanation, APOL1 variant cytotoxicity was proposed to result from APOL1 variant cationic pore-forming activity at the podocyte surface, increasing both Ca^2+^ and K^+^ fluxes (101, 102, 103, 105). Consistently, recent in silico studies suggested that K264 could directly occlude the APOL1 G2 pore (106, *Preprint*). However, this theoretical model conflicts with the strict requirement of acidic conditions for APOL1 transmembrane insertion (obviously absent during APOL1 secretion), and it does not consider the APOL1 wild-type-specific cis-interaction between C- and N-terminal domains (6, 24). Moreover, the proposed structural explanation for increased cation flux by G2 APOL1 does not apply to G1 APOL1 (106, *Preprint*). More importantly, this model clearly conflicts with the demonstration of highly conserved pore-forming activity of G2 N264K APOL1 in trypanosome endosomes (93). Conversely, podocyte dysfunctions underlying kidney disease can be induced independently of APOL1 pore-forming activity, such as occurs following APOL1 C-terminal truncation, which mimics G1 or G2 effects despite pore inactivation (6, 24). Finally, the pore-forming hypothesis does not explain why adsorption of APOL1 risk variants to lipid droplets (107) or depletion of cholesterol (108) both efficiently protect from kidney disease.

In summary, secreted APOL1 kills trypanosomes following transmembrane insertion in parasite endosomes, which is coupled to apoptotic-like permeabilization of the trypanosome mitochondrial membrane. APOL1 C-terminal variants that allow human resistance to *T. brucei rhodesiense* can cause kidney disease through podocyte dysfunctions linked to APOL3 inactivation. Under inflammatory conditions, this disease exhibits enhanced severity due to increased cationic fluxes at the podocyte surface, likely due to increased interaction of secreted APOL1 variants with cholesterol, which controls the activity of the podocyte TRPC6 and BK cation channels. In my view, this explanation is more probable than direct ion pore-forming activity of the APOL1 risk variants. Although such activity can be monitored in vitro for both recombinant APOL1 and APOL3, or in trypanosome endosomes for endocytosed APOL1, the basic physiological function of the flexible APOL transmembrane helices, flanked on both sides by fusogenic helices with potential cardiolipin-binding sites, appears to be promotion of membrane fusion (particularly cardiolipin solubilization), rather than ion fluxes. Thus, such ion fluxes would only be allowed under particular conditions, such as in vitro or outside of the normal cellular context, like in trypanosome endosomes (109). A similar conclusion can be proposed for the apoptotic BCL2 family proteins, which share with APOLs a double-stranded helix hairpin potentially able of transmembrane insertion. Indeed, the capacity of BCL2 proteins to form transmembrane ion pores in vitro appears to be irrelevant to their basic function as oligomeric megapore forming subunits (110).

During experimental infection of transgenic APOL1-expressing mice by the trypanosomatid parasites *Leishmania* major or *Leishmania amazonensis*, APOL1 was found to limit parasitemia through parasite damaging within the acidic parasitophorous vacuole (PV) of macrophages (111). Apart from this observation, there is, so far, no other demonstration of APOL1 involvement in parasite control. In particular, APOL1 does not affect infection by *Trypanosoma cruzi*, which unlike *T. brucei* exhibits intracellular development (111). Thus, secreted APOL1 appears to be a weapon specifically designed to act against bloodstream African trypanosomes. In strong support to this conclusion, secreted APOL1 acts in concert with a protein (HPR) that selectively allows efficient APOL1 targeting to African trypanosomes through interaction with a specific surface receptor of these parasites (90).

Like APOL1 in experimental *Leishmania* infection settings in mice, APOL3 or APOL3-like mAPOL7C may be involved in natural *Leishmania* infection control. Indeed, mAPOL7C is recruited to *Leishmania* major-containing PV membranes (42). Moreover, for its intracellular bactericidal activity, the mAPOL7C-like APOL3 exhibits synergistic activity with GBP1 (11, 74), a protein also recruited to PV membranes surrounding *S. typhimurium*, *Toxoplasma gondii*, *M. tuberculosis,* or *L. monocytogenes* (73, 75), where this protein controls pathogen growth through megapore formation in PV membranes and consecutive release of microbial molecules promoting inflammation (75). Thus, APOL3 and GBP1, two PI4P- and cardiolipin-binding proteins (6, 73, 76), could control not only intracellular bacterial growth but also *Leishmania* or *Toxoplasma* infection.

In conclusion, APOL1 and APOL3 are involved in the control of infection by bloodstream African trypanosomes and intracellular bacteria like *Salmonella*, respectively. However, given the synergistic activity of APOL3 with GBP1, the involvement of APOL3 in the control of other intracellular bacteria and parasites like *Leishmania *or *Toxoplasma* can be proposed. Thus, APOL3 mutations could be considered IEIs.

### APOLs’ roles in inflammation-linked increase of lipid droplets size

Murine mAPOL6 is specifically expressed in adipocytes, where it is involved in adipogenesis control (112, 113). The specific C-terminal extension of mAPOL6 promotes triglyceride accumulation through inhibition of lipolysis, due to direct interaction with perilipin-1 (Plin1), preventing Plin1 binding to the hormone-sensitive lipase involved in triglyceride degradation (112). Moreover, mAPOL6 controls the lipid droplet size during high-fat diet (113), notably through interaction with myosin-10 (MYO10) (20). Consistently, MYO10 governs both adipogenesis and PI4P-mediated lipid droplet dynamics (114). Interestingly, the mAPOL6 involvement in lipid droplet expansion coincides with the induction of low-grade inflammation in the adipose tissue, linking mAPOL6 activity to inflammation (113). Whether human APOL6 shares such activity is not known, but like mAPOL6, this isoform also contains a long C-terminal extension with both positively charged and highly hydrophobic stretches, which could inhibit lipolysis.

In conclusion, mAPOL6 appears to control inflammation-linked increase of adipocyte size during high-fat diet. Human APOL6 may exert a similar function. Given their capacity to interfere with APOL3 activity in human cells, the APOL1 G1 and G2 variants may similarly interfere with mAPOL6 activity in transgenic mice, accounting for their effects on diet-induced obesity (115, *Preprint*). Consistently, these variants also influence obesity in humans (116).

### APOLs’ involvement in inflammatory gene expression?

An entirely unexplored chapter deals with the enigmatic presence of APOLs, including APOL1 and APOL3, within the nucleus. Both APOLs are clearly detected in the nucleus in addition to the cytoplasm (6, 24), and APOL3 exhibits a putative bipartite nuclear localization signal possibly controlled by ubiquitination. Moreover, APOL1 immunoprecipitates are enriched in chromatin proteins and RNA-processing components (6). Therefore, at least some APOLs could shuttle between the cytoplasm and nucleus. The APOL3-interacting protein PI4KB, which like APOL3 harbors a bipartite nuclear localization signal, can also be found in the nucleus together with its product PI4P, where both components associate with mRNA speckles and factors involved in pre-mRNA splicing and/or transport (117, 118). Consistently, intranuclear lipid droplets can affect gene expression (119, 120, 121). Furthermore, myosins, known to associate with APOLs in the cytoplasm, can also be found in the nucleus, where they are involved in genome stability and gene expression (122). Thus, would the association of intranuclear APOLs with transcripts processing and trafficking factors influence gene expression through the control of mRNA editing and mRNA trafficking to the cytoplasm? If so, would APOLs participate in inflammation-linked gene expression changes?

## Conclusions

The APOL families are characterized by a dynamic evolution generating multiple isoforms with seemingly diverse functions, albeit largely linked to the control of pathogens. The basic function of APOLs appears to deal with membrane reorganization linked to immunity through association with both trafficking motors and fission/fusion factors.

Consistently, interference with APOLs activities may cause pleiotropic cellular dysfunctions linked to immunity. Natural C-terminal APOL1 mutations, which protect humans against deadly *T. brucei rhodesiense* infection, are responsible for APOL1-mediated kidney disease, but may also cause additional problems such as increased cardiovascular mortality, maternal preeclampsia, sepsis, neurotransmission disorders, cancer metastasis, and increased sensitivity to viral infection, such as increased nephropathy associated with HIV or COVID-19 infection (respectively HIVAN and COVAN, for HIV- and COVID-associated nephropathy) (123), all mainly attributable to APOL3 inactivation (81). The evolutionary selection of these two deleterious mutations, and their large spreading in Western Africa, is likely to have resulted from their capacity to confer resistance to *T. brucei rhodesiense* infection, presumably leading to the disappearance of this parasite from this part of the continent (88). Natural null mutations of APOL3 also induce kidney disease (94, 95), probably together with immunity problems given the basic cellular dysfunctions observed in cultured APOL3 KO podocytes (6, 24). However, presumably given the absence of APOL3 protective effect against trypanosome infection, these mutations are rarely detected. Natural null mutations of APOL1, identified in a single occasion in India, not only induce the loss of resistance against *Trypanosoma evansi* infection (92) (Fig. 5) but are also expected to induce other dysfunctions of the immune system, notably impairment of mitophagy and apoptosis as observed in cultured APOL1 KO cells (6, 24). This explains why natural APOL1 mutations are considered IEIs (124), but rare natural APOL3 mutations and eventual natural mutations in other APOL isoforms could be added to the list.

Many issues remain to be clarified, including the following:

• How does IFN-I trigger PI4KB and ARF1 traffic in ATG9A vesicles from the Golgi to MERCS: is ARF1 activation responsible for PI4KB dissociation from APOL3, mimicking APOL3 downregulation?
• How is APOL1 associated with the NM2A myosin (and mAPOL6 with the MYO10 myosin)? Would APOL1 interact with the RLC of NM2A, like APOL3 interacts with NCS1, which shares with RLC a similar calmodulin-like structure?
• Is APOL1 involved in NM2A activity through RLC dissociation from the myosin heavy chain, as occurs following NM2A interaction with anionic phospholipids (125) or MYO6 interaction with cardiolipin (69)?
• Are APOL1 and APOL3 involved in STING activation and degradation?
• Is APOL3 associated with STING and/or GOLPH3?
• How do APOL isoforms target different membranes?
• What is the mechanism of membrane permeabilization by mAPOL7C or APOL3? Are members of the apoptotic BCL2 family involved in this process?
• Is APOL3 involvement in intracellular bacterial membrane permeabilization related to APOL3-mediated permeabilization of phagolysosomal membranes?
• Is APOL3 involvement in intracellular bacterial membrane permeabilization related to APOL3-mediated mitochondrial membrane fusion?
• Could the increase of interaction with cholesterol be responsible for the inflammation-linked cytotoxicity of APOL1 risk variants that cause APOL1-mediated kidney disease?
• Is human APOL6 activity restricted to adipogenesis control in adipocytes, as it occurs for mAPOL6 in mice?
• What is the role of intranuclear APOLs? Is their inflammation-driven accumulation in the nucleus linked to gene expression changes for immunity development?
